# Machine Learning Models and Pathway Genome Data Base for *Trypanosoma cruzi* Drug Discovery

**DOI:** 10.1371/journal.pntd.0003878

**Published:** 2015-06-26

**Authors:** Sean Ekins, Jair Lage de Siqueira-Neto, Laura-Isobel McCall, Malabika Sarker, Maneesh Yadav, Elizabeth L. Ponder, E. Adam Kallel, Danielle Kellar, Steven Chen, Michelle Arkin, Barry A. Bunin, James H. McKerrow, Carolyn Talcott

**Affiliations:** 1 Collaborative Drug Discovery, Burlingame, California, United States of America; 2 Collaborations in Chemistry, Fuquay-Varina, North Carolina, United States of America; 3 Skaggs School of Pharmacy and Pharmaceutical Sciences, University of California, San Diego, San Diego, California, United States of America; 4 SRI International, Menlo Park, California, United States of America; 5 Chemistry, Engineering & Medicine for Human Health (ChEM-H), Stanford, California, United States of America; 6 Department of Pathology, University of California, San Francisco, San Francisco, California, United States of America; 7 Small Molecule Discovery Center and Department of Pharmaceutical Chemistry, University of California, San Francisco, San Francisco, California, United States of America; University of Washington, UNITED STATES

## Abstract

**Background:**

Chagas disease is a neglected tropical disease (NTD) caused by the eukaryotic parasite *Trypanosoma cruzi*. The current clinical and preclinical pipeline for *T*. *cruzi* is extremely sparse and lacks drug target diversity.

**Methodology/Principal Findings:**

In the present study we developed a computational approach that utilized data from several public whole-cell, phenotypic high throughput screens that have been completed for *T*. *cruzi* by the Broad Institute, including a single screen of over 300,000 molecules in the search for chemical probes as part of the NIH Molecular Libraries program. We have also compiled and curated relevant biological and chemical compound screening data including (i) compounds and biological activity data from the literature, (ii) high throughput screening datasets, and (iii) predicted metabolites of *T*. *cruzi* metabolic pathways. This information was used to help us identify compounds and their potential targets. We have constructed a Pathway Genome Data Base for *T*. *cruzi*. In addition, we have developed Bayesian machine learning models that were used to virtually screen libraries of compounds. Ninety-seven compounds were selected for *in vitro* testing, and 11 of these were found to have EC_50_ < 10μM. We progressed five compounds to an *in vivo* mouse efficacy model of Chagas disease and validated that the machine learning model could identify *in vitro* active compounds not in the training set, as well as known positive controls. The antimalarial pyronaridine possessed 85.2% efficacy in the acute Chagas mouse model. We have also proposed potential targets (for future verification) for this compound based on structural similarity to known compounds with targets in *T*. *cruzi*.

**Conclusions/ Significance:**

We have demonstrated how combining chemoinformatics and bioinformatics for *T*. *cruzi* drug discovery can bring interesting *in vivo* active molecules to light that may have been overlooked. The approach we have taken is broadly applicable to other NTDs.

## Author Summary

Chagas disease is a neglected tropical disease (NTD) caused by the eukaryotic parasite *Trypanosoma cruzi*. The disease is endemic to Latin America but is increasingly found in North America and Europe, primarily through immigration, and the spread of this disease is bringing new attention to the need for novel, safe, and effective therapeutics to treat *T*. *cruzi* infection. We have used data from a phenotypic screen to build Bayesian models to predict anti-parasitic activity against *T*. *cruzi in vitro*. These models were used to score various small libraries of molecules. We selected less than 100 compounds for testing and found *in vitro* actives, some of which were tested in an *in vivo* efficacy model. We identified the antimalarial pyronaridine as having *in vivo* efficacy and provides us with a new starting point for further investigation and optimization.

## Introduction

In the 1980’s the pharmaceutical industry took advantage of advances in molecular biology/genetic engineering and began replacing phenotypic, whole-cell HTS with target-based screening assays [1]. Target-based screens using simple recombinant protein enzymatic assays offer advantages in terms of cost and scalability. Nonetheless, in the last decade, there has been a shift back towards using phenotypic screens as a starting point for drug discovery, especially for infectious diseases where drug targets are poorly understood or target-based approaches have been unsuccessful in the past [1]. In fact, analysis of the origin of first-in-class small molecules found that phenotypic screens identified more novel inhibitors than any other approach between 1999 and 2008 [2,3].

One such disease area, where target-based drug discovery has largely failed, is in the field of neglected tropical diseases (NTDs). NTDs are a collection of infectious diseases that disproportionately affect marginalized or poor populations in the developing world [4]. Many of these pathogens are eukaryotic parasites with complex life cycles and diverse approaches for evading the host immune system. Furthermore, many of these parasites are not genetically tractable in the laboratory and receive only a small amount of research investment from scientists and pharmaceutical companies in the developed world [5]. The trend towards using phenotypic screens over target-based screens is particularly strong for NTDs as well as bacterial and fungal pathogens. For these infectious diseases, it is generally considered more difficult to convert a strong targeted hit into a cell permeable, non-toxic drug than it is to identify the target of a non-toxic compound with phenotypic, whole-cell activity [6], especially in the case of intracellular parasites in which the compound has to cross an extra membrane of the host cell to hit its final target.

Chagas disease is an NTD caused by the eukaryotic parasite *Trypanosoma cruzi* [7]. The disease is endemic to Latin America but is increasingly found in North America and Europe, primarily through immigration [8–11] and the spread of this disease is bringing new attention to the need for novel, safe, and effective therapeutics to treat *T*. *cruzi* infection. The current clinical and preclinical pipeline for *T*. *cruzi* is extremely sparse and lacks drug target diversity (currently focused on 3 targets, CYP51, cruzain and genes associated with DNA damage) [12–14]. Pre-clinical development of oxaboroles is being led by a partnership between DNDi and Anacor [15]. The most advanced product is the re-evaluation of a toxic general DNA damage agent benznidazole, approved for use in Chagas disease outside the U.S but not by the US FDA. It requires dosing of sixty days or more and has significant toxicity [16,17]. The remaining products in clinical development (Phase I and II) target a single enzyme, CYP51, which has been the focus of Chagas disease drug development to date [18–23]. Recent results from Phase II trials demonstrated that repurposed drugs targeting fungal CYP51 did not eliminate recrudescent parasites at 6 months post therapy as determined by PCR [24]. Attention has therefore shifted to drug development targeting the parasite CYP51 itself [20,22] such as fexinidazole [25,26]. The only additional novel drug target with a single compound in preclinical development is cruzain, a *T*. *cruzi* cysteine protease and there is considerable literature surrounding this class of inhibitors [27,28] as well as overlap with CYP51 [29].

There have been some target-based high throughput screens for inhibitors of CYP51 [23] and cruzain [28] as well as virtual screening of inhibitors for cruzain [27]. Several whole-cell, phenotypic high throughput screens have been completed for *T*. *cruzi*, including most recently a screen of 1.8 million compounds at GlaxoSmithKline in Spain [30], another of over 300,000 molecules at the Broad Institute [31–34] and a proprietary screen by the Genomics Institute of the Novartis Research Foundation (GNF) [35]. Therefore more HTS is leading to new hits [31–39] from academia [40], industry, and the non-profit sector, primarily with the support of NIAID and the Drugs for Neglected Diseases Initiative (DNDi). However, there is a disconnect between the currently identified targets and outcomes obtained in clinical trials [41]. The latest HTS hits are also early in the pipeline. Methods for identifying and prioritizing novel targets of phenotypic screening hits will become increasingly important as well as approaches to screen vast libraries of molecules using computational approaches prior to *in vitro* testing.

In the past we have used a used a combined bioinformatics-cheminformatics approach to compile, analyze, and prioritize novel metabolic enzyme targets from *Mycobacterium tuberculosis (Mtb)*, then suggest compounds that might interact with these targets [42]. One study identified 12 enzymes that are *in vivo* essential enzymes in *Mtb*, absent in humans, have known reactions in TBCyc (http://tbcyc.tbdb.org/index.shtml; an *Mtb*-specific metabolic pathway database), and are not targets of known TB drugs. These targets and their metabolites were used with a 3D pharmacophore approach to screen vendor libraries [43–45] before filtering with additional computational models [43,46,47]. Ultimately novel inhibitors were identified showing moderate minimal inhibitor concentration values against *M*. *tuberculosis in vitro* [42]. These are currently undergoing further validation. In contrast to tuberculosis, there are significantly fewer public, curated, and compiled data on metabolic pathways and computational drug screening efforts in *T*. *cruzi* [48–50].

In the current study we have compiled and curated relevant biological and chemical compound screening data including (i) compounds and biological activity data from the literature, (ii) high throughput screening datasets, and (iii) predicted metabolites of *T*. *cruzi* metabolic pathways. To this end, we identified and extracted associated biological data for 584 compounds with activity data against *T*. *cruzi* in the published literature and made this available as a public dataset in CDD Public. In addition we have created a BioCyc database for *T*. *cruzi*, which complements other sources of related metabolic pathway data (including KEGG *T*. *cruzi* pathways [51], BioCyc databases for the closely related pathogens *Leishmania major* [52] and *Trypanosoma brucei* [28], and the PathCase Metabolic Workbench dataset for *T*. *cruzi* [53]) and can be used in future drug discovery efforts. We have also compiled public screening data for the over 300,000 additional compounds screened against *T*. *cruzi* and the related pathogen *Trypanosoma brucei* [54,55]. Subsets of these data have been used to build machine learning models for compound selection as we have previously done with *Mtb* datasets [43,46,47,56–61]. All of these efforts and curated information on *T*. *cruzi* may be used for target inference [62,63] which combines cheminformatics and bioinformatics capabilities. Ultimately we highlight how our approach lead to *in vivo* testing of compounds and the discovery of a promising lead candidate.

## Methods

### CDD database and Chagas datasets

An analysis of the Chagas disease literature was performed resulting in the curation of over 500 molecules with associated target information (when available). The Broad Chagas screening data [31–34] were also collected and both datasets were uploaded into the CDD database (Collaborative Drug Discovery Inc. Burlingame, CA) [64] from sdf files and mapped to custom protocols [65]. All public datasets used in model building are available for free public read-only access and mining upon registration in the CDD database [66]. The Broad dataset (TRYPANOSOME: Broad Primary HTS to identify inhibitors of T. Cruzi Replication) used in this study is also available in PubChem (AID 2044). In addition we curated Chagas compounds from the literature and made these public (TRYPANOSOME: Chagas Disease Literature Compounds).

### Data annotation and Pathway Genome Data Base construction

By using a combination of genetic validation from the literature, bioinformatic analyses, and available assays, we prioritized *T*. *cruzi* targets for experimental validation as the binding targets of screening hits. Furthermore, SRI has developed “choke point” analyses to assess the likelihood that a particular metabolic pathway step is essential for an organism [67,68]. In order to use such approaches we constructed a Pathway Genome Data Base (PGDB) for *T*. *cruzi* (which we coined as “TCruCyc”) using the complete genome sequence of the Dm28c strain. The Dm28c strain was chosen over the more common CL-Brener strain since it is a model organism for studying Chagas disease and its recently assembled genome sequence [69] is more complete than CL-Brener (whose repeat sequences have hindered complete assembly). This was completed by using the “Pathologic” workflow within the Pathway Tools suite [70,71]. The existing workflow imports the complete genome sequence and then assigns proteins from annotated sequences. A patch to Pathologic to enable proteins to be searched by Uniprot/TrEMBL identifiers was used. This process will not assign proteins unless they are annotated in the genome sequence, which will miss some obvious sequence-based homologies (e.g. the tubulin gene is not annotated in the Dm28c sequence). We also explored workflows that would enable the automatic import of protein annotations from a closely related organism (e.g. CL-Brener), but ended up manually annotating a number of orphan proteins for our current dataset. The underlying genome sequence consisted of 5,287 contigs assembled into 1,378 scaffolds of 30,716,540 base pairs. Pathologic found 11,349 distinct gene products, at least 880 of which were found to be enzymes and at least 16 of which are transporters. Pathologic was able to infer 1030 enzymatic reactions and 122 pathways from these assignments as well as the existence of 806 metabolic compounds. This set was filtered to 358 molecules after removal of compounds with R- groups and small nuisance molecules. This dataset was then used to infer potential targets by comparing the Tanimoto similarity with a phenotypic screening hit [42]. The *T*. *cruzi* PGDB can be accessed at http://node2.csl.sri.com:1555/.

### Building and validating dual-event machine learning models with novel bioactivity and cytotoxicity data

In our previous publications we have described the generation and validation of the Laplacian-corrected Bayesian classifier models developed with bioactivity and cytotoxicity data to create dual-event models [72–74] using Discovery Studio versions 3.5 and 4.1 (Biovia, San Diego, CA) [75–79]. We have now applied this approach to the Broad Chagas dose response data (AID 2044) [31–33] using the EC_50_ data, where values less than 1 μM are classed as actives and were used for the single event models. We further refined the actives using the cytotoxicity data when a greater than 10 fold difference with cytotoxicity was observed and these compounds were considered active. The models were all generated using the following molecular descriptors: molecular function class fingerprints of maximum diameter 6 (FCFP_6) [80], AlogP, molecular weight, number of rotatable bonds, number of rings, number of aromatic rings, number of hydrogen bond acceptors, number of hydrogen bond donors, and molecular fractional polar surface area which were all calculated from input sdf files.

The resulting single- and dual-event datasets were validated using leave-one-out cross-validation, 5 fold validation and by leaving out 50% of the data and rebuilding the model 100 times using a custom protocol to generate the receiver operator curve area under the curve (ROC AUC), concordance, specificity and selectivity as described previously [72–74].

These models were used to score the following drug libraries; Selleck Chemicals (Houston, TX) natural product library (139 molecules), GSK kinase library (367 molecules) [81], Malaria box (400 molecules) [82], Microsource (Gaylordsville, CT) Spectrum (2320 molecules), CDD FDA drugs (2690 molecules), Prestwick Chemical (Illkirch, France) library (1280 molecules) and Traditional Chinese Medicine components (373 molecules, kindly provided by Dr. Ni Ai, Zhejiang University, China). The top scoring molecules with the dual event model were selected and purchased from eMolecules (La Jolla, CA) and then 97 underwent primary *in vitro* screening.

### Primary *in vitro* screening

Mouse myoblast cell line C2C12 (ATCC #CRL-1772) was cultivated in Dulbecco’s Modified Eagle’s Medium containing 4.5 g/l glucose (DMEM), supplemented with 5% fetal bovine serum (FBS), 25 mM HEPES, 2 mM L-glutamine, 100 U/ml penicillin and 100 μg/ml streptomycin. *T*. *cruzi* CA-I/72 trypomastigotes were obtained from C2C12 infected-culture supernatants after 4–7 days of infection. Cultures were maintained at 37°C with 5% CO_2_. For the infection assay to assess anti-parasitic activity of the compounds, 500 C2C12 cells were seeded in 384-well plate in 40 μl of DMEM media per well. Compounds were added at 10 mM in 50 nl per well using a Biomek FX (Beckman Coulter) for a final 10 μM concentration in 50 μl total volume, and 2,500 parasites were added in 10 μl per well. The plate was incubated for 72 hours at 37°C with 5% CO_2_. After the incubation, the plate was fixed with the addition of 50 μl of 8% paraformaldehyde solution, followed by two successive washing steps using PBS. Finally, a staining solution containing 0.5 μg/ml of 4',6-diamidino-2-phenylindole (DAPI) was added to each well of the plate and incubated for at least 4 hours prior to reading. Images were acquired by an IN Cell Analyzer 2000 (GE Healthcare) and analyzed by IN Cell Analyzer Developer 1.6 software. The size parameters used to segment host and parasite organelles were 125 μm^2^ for host nucleus, and 1–2 μm^2^ for parasite nucleus/kinetoplast. Numbers of host cells and intracellular amastigotes were determined based on host cell and parasite nucleus quantification, providing a measure of growth inhibition during the first 72 h of post-infection treatment compared to untreated controls. The anti-parasitic results were expressed in terms of relative activity normalized based on the average infection ratio (number of infected cells/total number of cells) of negative controls (0.1% DMSO, 0% activity) and positive controls (50 μM of benznidazole, EC_100_, 100% activity). The host cell viability was assessed based on the total number of cells divided by the average number of cells from untreated controls (0.1% DMSO), being <0.5 considered a cytotoxic compound. This assay was performed in duplicate.

### Hit selection and secondary screening (dose-response assay)

The hit selection criteria: >50% activity at 10 μM and >0.5 host cell viability in the primary screening. To determine the potency of the hit compounds, we performed a dose-response assay. EC_50_ values of compounds were determined applying the same assay used in the primary screening. For this, an intermediate plate (384-well plate) was prepared by serial diluting each hit compound (10 mM, 5 mM, 2.5mM, 1.125 mM, 0.625 mM, 0.312 mM, 0.156 mM, 78 μM, 36 μM, 18 μM) in 100% DMSO. Then, 50 nl of each sample were diluted in 50 μl media (DMEM H-21) and added to the experimental plate followed by incubation at 37°C with 5% CO_2_ for 72 h.

### *In vivo* studies

To assess *in vivo* efficacy of test compounds, a 4-day mouse model of infection by transgenic *T*.*cruzi* Brazil luc strain expressing firefly luciferase was used as previously described [83]. Six-week-old female Balb/c mice (average weight 20g) were obtained from Simonsen Labs (Gilroy, CA). All animal protocols were approved and carried out in accordance with the guidelines established by the Institutional Animal Care and Use Committee from UCSD (Protocol S14187). Mice were housed at a maximum of 5 per cage and kept in a specific-pathogen-free (SPF) room at 20 to 24°C under a 12-h light/12-h dark cycle and provided with sterilized water and chow ad libitum. To infect the mice, trypomastigotes of *T*. *cruzi* Brazil luc strain were used. The parasites were harvested from culture supernatant 7 days after the infection of C2C12 myocytes in T.75 culture flasks using DMEM media supplemented with 5% FBS. The harvested parasites were counted and the density was adjusted for 10^6^ parasites per milliliter of DMEM media without FBS. For the mouse infection, 100 ul of the parasite solution was injected intraperitoneally (10^5^ trypomastigotes) per mouse. Starting on day 3 the infected mice were treated with test compounds at 50 mg/kg administered in 20% Kolliphor, IP, b.i.d., for four consecutive days. Two control groups included untreated mice, which received a vehicle (20% Kolliphor HS 15, a.k.a. Solutol), and the positive control groups, which received 50 mg/kg benznidazole, IP, twice a day (b.i.d). At day 7 post-infection, the luminescent signal from infected mice was read upon injection of D-luciferin. The absolute numbers of measured photons/s/cm^2^ were averaged between all five mice in each group. The average photons/s/cm^2^ from the group treated with benznidazole was normalized as 100% efficacy and the average photons/s/cm^2^ from the group treated with vehicle only was normalized as 0% efficacy. Using a linear correlation, the average photons/s/cm^2^ of each compound was normalized in the same efficacy scale as the controls.

### Statistics

Two tailed paired Student *t* test was used to verify the hypothesis that the luminescence values from vehicle-treated and compound-treated groups at day 7 post-infection were significantly different (p≤ 0.05).

## Results

### Data annotation and Pathway Genome Data Base construction

**Fig 1:**
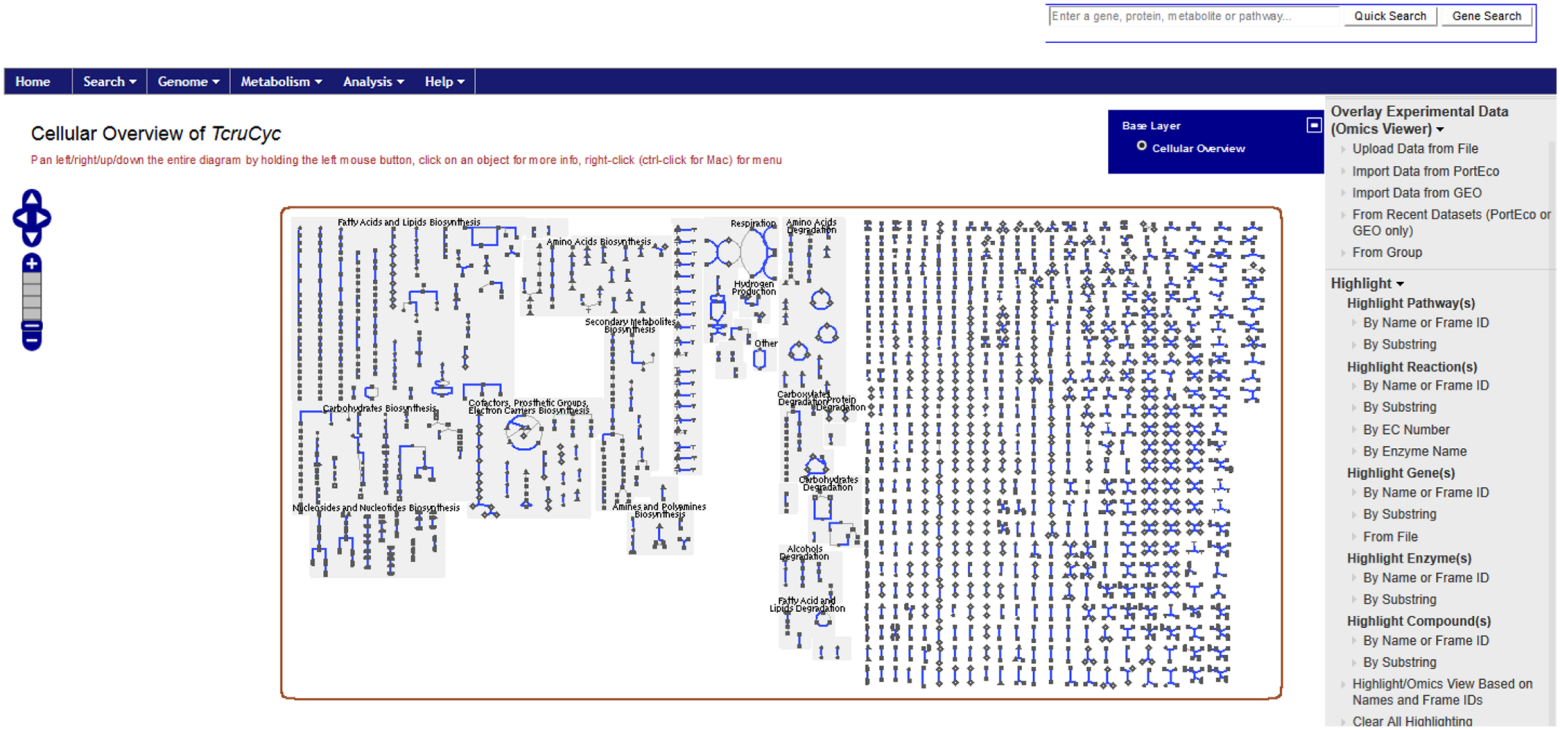
A typical metabolic cellular overview of TCruCyc provided by the Pathway Tools web server. This view of the TCruCyc PGDB shows the (almost entirely) inferred set of metabolic pathways from gene sequence data. Canonical pathways such as “Amino Acids Biosynthesis”, “Amino Acids Degradation”, “Nucleosides and Nucleotides Biosynthesis”, “Fatty Acids and Lipids Biosynthesis” and “Respiration” are partially inferred as well as a large set of single reaction steps (right side) that Pathway Tools could integrate into larger pathways. This is an expected level of derivable connectivity that would be available from annotated genome and proteome sequence data. We expect that a significant number of unassigned protein functions can be assigned by extending Pathway Tools with (high threshold) automated sequence similarity analysis that is currently done via manual curation.

A PGDB was constructed for *T*. *cruzi* using the complete genome sequence of the Dm28c strain (Fig 1). The underlying genome sequence consisted of 5,287 contigs assembled into 1,378 scaffolds of 30,716,540 base pairs. Pathologic found 11,349 distinct gene products, at least 880 of which were found to be enzymes and at least 16 of which are transporters. Pathologic was able to infer 1030 enzymatic reactions and 122 pathways from these assignments as well as the existence of 806 metabolic compounds. This set was filtered to 358 molecules after removal of compounds with R- groups and small nuisance molecules. This dataset was then used to infer potential targets by comparing the Tanimoto similarity with a phenotypic screening hit [42].

### Bayesian models

Using either dose response data alone (S1 Dataset) or the combination of dose response and cytotoxicity (dual activity, S2 Dataset) resulted in statistically comparable models. Both had leave one out Receiver Operator Curve (ROC) values greater than 0.8 (Table 1). The use of FCFP_6 fingerprints enabled the features important for activity (termed good features) to be visualized in the dose response data alone model (S1 Fig) which included tertiary amines, piperidines and aromatic fragments containing basic nitrogen functionality while those features that were negatively related to activity included cyclic hydrazines prone to tautomerization as well as a number of electron-poor chlorinated aromatic systems (S2 Fig). Similarly for the dual activity the good features were tertiary amines, piperidines and aromatic fragments containing basic nitrogen functionality (S3 Fig) and the bad features were again a number of cyclic hydrazines prone to tautomerization and a number of electron-poor chlorinated aromatic systems (S4 Fig) Upon 5 fold cross validation the ROC was greater than 78% for both models and sensitivity, specificity and concordance values were comparable and greater than 77% (Table 1). The more exhaustive leave out 50% x 100 fold for the dual activity model resulted in an external ROC of 0.79 and while concordance and specificity was greater than 73%, sensitivity declined to 66% (S1 Table).

**Table 1:** Leave-out cross validation data for *T*.*cruzi* Bayesian models.

Model	Best cutoff	Leave-one out ROC	5-fold cross validation ROC	5-fold cross validation sensitivity (%)	5-fold cross validation specificity (%)	5-fold cross validation concordance (%)
Dose response (1853 actives, 2203 inactives)	-0.676	0.81	0.78	77	89	84
Dose response and cytotoxicity (1698 actives, 2363 inactives)	-0.337	0.82	0.80	80	88	84

### *In vitro* screening

Approximately 7200 molecules were screened using the Bayesian model. Molecules with the highest Bayesian score in the dual event model were selected by an experienced medicinal chemist and purchased. Ninety seven molecules were tested and 11 were found to have EC_50_ values less than 10μM (S2 Table). Five of these molecules (verapamil, pyronaridine, furazolidone, tetrandrine and nitrofural) had *in vitro* EC_50_ values less than 1μM (Table 2).

**Table 2:** *In vitro* and *in vivo* data for compounds selected in this study.

Synonyms	Infection Ratio	EC_50_ (μM)	EC_90_ (μM)	Hill slope	Cytotoxicity CC_50_ (μM)	Chagas mouse model (4 days treatment, luciferase): *In vivo* efficacy at 50 mg/kg bid (IP) (%)
(±)-Verapamil hydrochloride, 715730, SC-0011762	0.02, 0.02	0.0383	0.143	1.67	>10.0	55.1
29781612, Pyronaridine	0.00, 0.00	0.225	0.665	2.03	3.0	85.2
511176, Furazolidone	0.00, 0.00	0.257	0.563	2.81	>10.0	100.5
501337, SC-0011777, Tetrandrine	0.00, 0.00	0.508	1.57	1.95	1.3	43.6
SC-0011754, Nitrofural	0.01, 0.01	0.775	6.98	1.00	>10.0	78.5*

* Used hydroxymethylnitrofurazone for *in vivo* study (nitrofural pro-drug)

### *In vivo* testing

**Fig 2:**
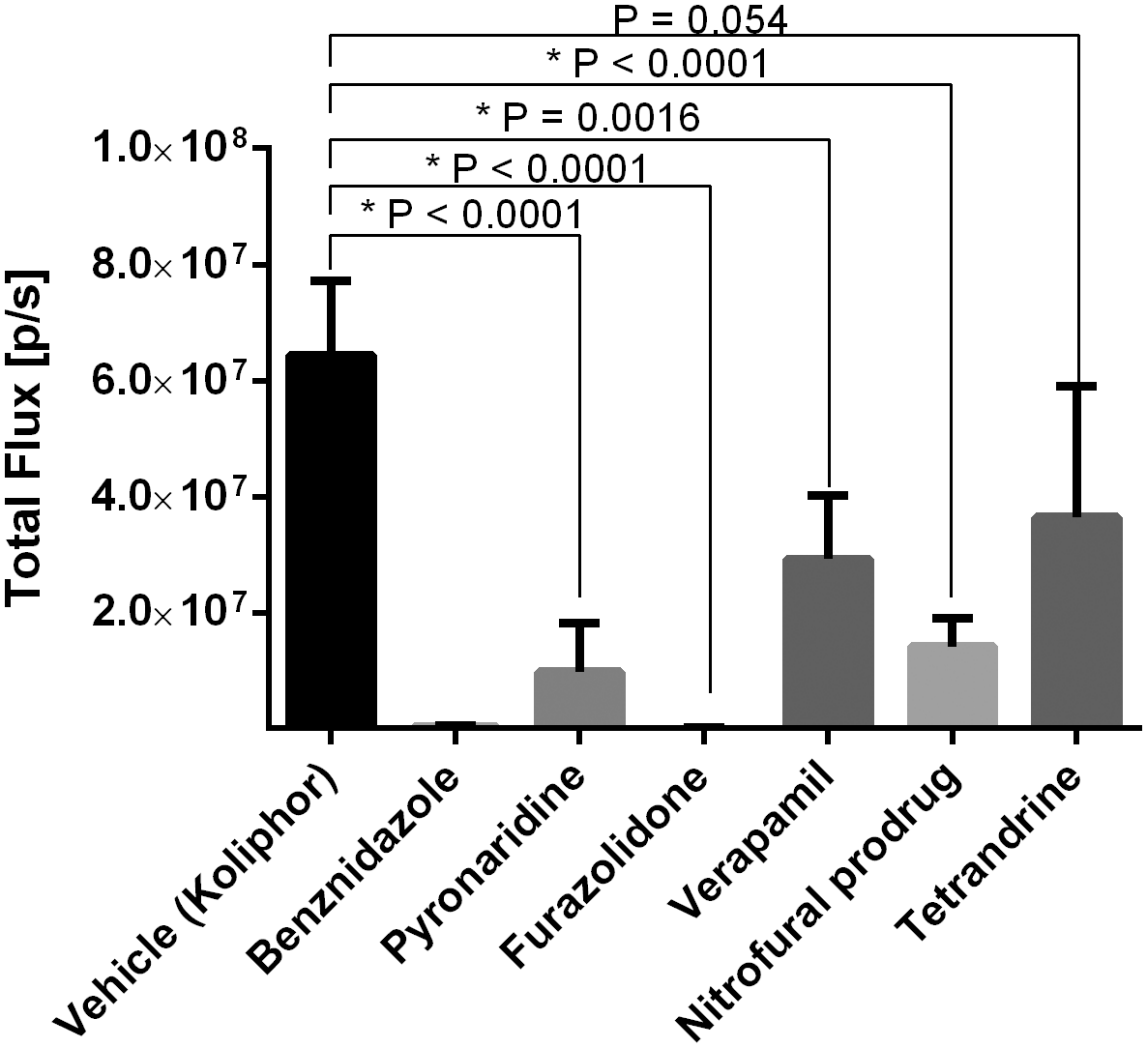
*In vivo* efficacy of test compounds (50mg/kg b.i.d.) in a 4-day mouse model of infection by transgenic *T*.*cruzi* Brazil luc strain35 expressing firefly luciferase.

To assess *in vivo* efficacy of test compounds, a 4-day treatment mouse model of infection by transgenic *T*.*cruzi* Brazil luc strain35 expressing firefly luciferase was used [83] which enabled the activity in the mouse to be visually measured (S5 Fig). All compounds were dosed at 50mg/kg bid. Benznidazole was used as a positive control and showed 100% efficacy alongside furazolidone (Fig 2 and Table 2). Hydroxymethylnitrofurazone is a prodrug of nitrofural (which had *in vitro* activity) and is an additional known active compound against Chagas Disease, with an efficacy of 78.5%. We chose the prodrug form to reduce the toxicity of nitrofural in the mouse model [84]. Pyronaridine showed 85.2% efficacy while verapamil showed 55.1% and tetrandrine 43.6%, respectively. Apart from tetrandrine, these are statistically significant (Fig 2 and Table 2).

### Target prediction

Using several available datasets and resources we investigated the potential target/s of pyronaridine. First we performed a similarity search in the Chagas Disease dataset composed of literature data and targets which was curated in this study. The molecules with the highest Tanimoto similarity in CDD were *T*. *cruzi* GAPDH inhibitors (S6 Fig). We also searched the metabolites created from the *T*. *cruzi* pathway model created in this study. The most similar molecule being S-adenosyl 3-(methylthio)propylamine with a Tanimoto similarity of 0.67 using the MDL Keys in Discovery Studio (Biovia, San Diego, CA). This would point to polyamine biosynthesis [85]. A further approach was to query the ChEMBL database from within the MMDS mobile app (S7 Fig). This retrieved several analogs similar to the antimalarial quinacrine, suggesting trypanothione disulfide reductase [86,87] as a possible target. Quinacrine has also been shown to be a Topoisomerase VI inhibitor elsewhere [88]. These targets will be evaluated in future studies to identify whether they have a role in the mechanism of action of pyronidine in *T*. *cruzi*.

## Discussion

Our prior computational drug discovery work in *Mycobacterium tuberculosis* [42] was made possible by the existence of datasets with genetic validation of essential genes *in vivo*. The work profited from the existence of the tier one TBCyc metabolic pathway database, the natural divergence of prokaryotic *M*. *tuberculosis* genome from the genome of the eukaryotic human host, and the availability of a well-annotated *M*. *tuberculosis* genome [24,34]. In contrast, *T*. *cruzi*, the eukaryotic parasite that causes Chagas disease, and several other eukaryotic human pathogens including the parasites that cause malaria, human African trypanosomiasis, and leishmaniasis, have larger genomes, higher similarity to human enzymes and biological pathways, and have less well annotated genomes. Investment in high throughput screening efforts has resulted in the release of screening data and hit lists for several of these eukaryotic pathogens [35–36]. However, identification of targets of hit compounds has seen relatively slow progress. Therefore, we hypothesized that for pathogens, such as *T*. *cruzi*, with fewer sources of available data to support bioinformatics approaches to target identification, we can take a reverse approach as compared to our work in *Mycobacterium tuberculosis*. More specifically, we can start with interesting phenotypic screening hits and apply cheminformatic and bioinformatic approaches to map those hits onto potential targets. As a preliminary step in this direction we have used public data to build computational models.

The CDD Public database now includes structural and biological activity data for over 300,000 molecules from the Broad Institute compounds that have been screened against *T*. *cruzi*. In addition we have curated over 500 compounds and their known targets and over 740 compounds from DNDi based around the fungicide fenarimol, as separate datasets. In this study, we have utilized a subset of the Broad HTS screening data to build Bayesian machine learning models to classify compounds as likely actives against *T*. *cruzi in vitro*. We then used these models to virtually screen several libraries of compounds including drugs and drug-like compounds, to identify compounds with potential activity that may have not been tested yet. Some of these compounds were purchased and tested *in vitro* and then several more tested *in vivo*. Historically, for a diversity-based library undergoing HTS, it is expected a range of 1 to 2% of hits based on observed activity (usually >50% antiparasitic activity at 10 μM and no signs of cytotoxicity at this concentration) will be observed [34]. Applying the current method, 11/97 (11%) hits were identified and confirmed with EC_50_ < 10 μM.

Out of these hits derived from searching 8 relatively small libraries of compounds, several of the compounds were found to be known actives against *T*. *cruzi*. Verapamil was previously shown as active in the Broad dataset with an EC_50_ < 0.1μM, and has a well-known effect in reducing acute mortality in mice [89,90] and cardiomyopathy if treated early in infection [91]. It should be noted that others have retested some of the active HTS hits from the Broad *T*. *cruzi* screen and found higher IC_50_ values. For example the IC_50_ for verapamil in one study was >50 μM [38]. Pyronaridine is in clinical use as an antimalarial [92,93], is a P-glycoprotein inhibitor [94] and was given a positive opinion by the European Medicines Agency using this molecule in a combination therapy [95]. It was shown to have an EC_50_ < 0.587μM in the Broad dose response dataset, which is comparable to this study (EC_50_ 0.225 μM). Apparently both of these compounds were retrieved as various salt forms from the vendor databases and were initially not considered to be in the training sets. Pyronaridine as far as we can tell, was overlooked following the published initial screening [34] and so we pursued these compounds further *in vivo*. Furazolidone is used as a *H*. *pylori* treatment [96] and has known *in vivo* activity against *T*. *cruzi* [97] and was not in the dose response training set (but is in the larger Broad screening dataset of over 300,000 compounds), so can be considered a true ‘prediction’. Tetrandrine is a P-glycoprotein inhibitor [98] that has been tested in malaria in combination with chloroquine [99]. This molecule was not in the training dataset but was in the larger Broad HTS screening dataset to identify inhibitors of replication as an ‘inactive’, so our ability to identify a previous false negative as an active prediction is an interesting observation, although this compound does not appear to have statistically significant efficacy *in vivo*. The known *T*. *cruzi* active compound Nitrofural (nitrofurazone) [97] was also not in the model training set or the Broad dataset, but was predicted as ‘active’ *in vitro* (experimentally confirmed EC_50_ 0.77μM and CC_50_ > 10μM), and its prodrug form hydroxymethylnitrofurazone was used as an internal control (while benznidazole was a positive control) in the *in vivo* experiments. These results illustrate that the dose response and cytotoxicity machine learning model based on *T*. *cruzi* replication HTS data [34] used in this case, could retrieve known active compounds useful for Chagas Disease. While the Broad screen and the assay used in this study are similar in that they are both cell-based, they each use different cell lines for *T*. *cruzi* culture and different readouts. The Broad screen used the Tulahuen genetically modified to express Beta-galactosidase [34,54] which is biased towards finding CYP51 inhibitors [35], while we used the CA-I/72 strain with an image-based readout. We are not aware of publications describing pyronaridine being tested in the mouse model for Chagas disease and our observation of 85.2% efficacy (higher than nitrofural) suggests this molecule is therefore worthy of further study (Fig 2 and S5 Fig). In particular, the identification of the likely target or targets for this molecule would be very important. Using various informatics resources we have attempted to predict these in this study. Our prior work on *Mtb* resulted in many datasets relating to small molecules and their targets in the bacteria, which in turn lead to the development of the TB Mobile app which contains Bayesian models that can be used for target prediction [56,62,63]. While we do not have as much published data for *T*. *cruzi* a similar approach could be undertaken in future for target prediction in NTDs more broadly.

**Fig 3:**
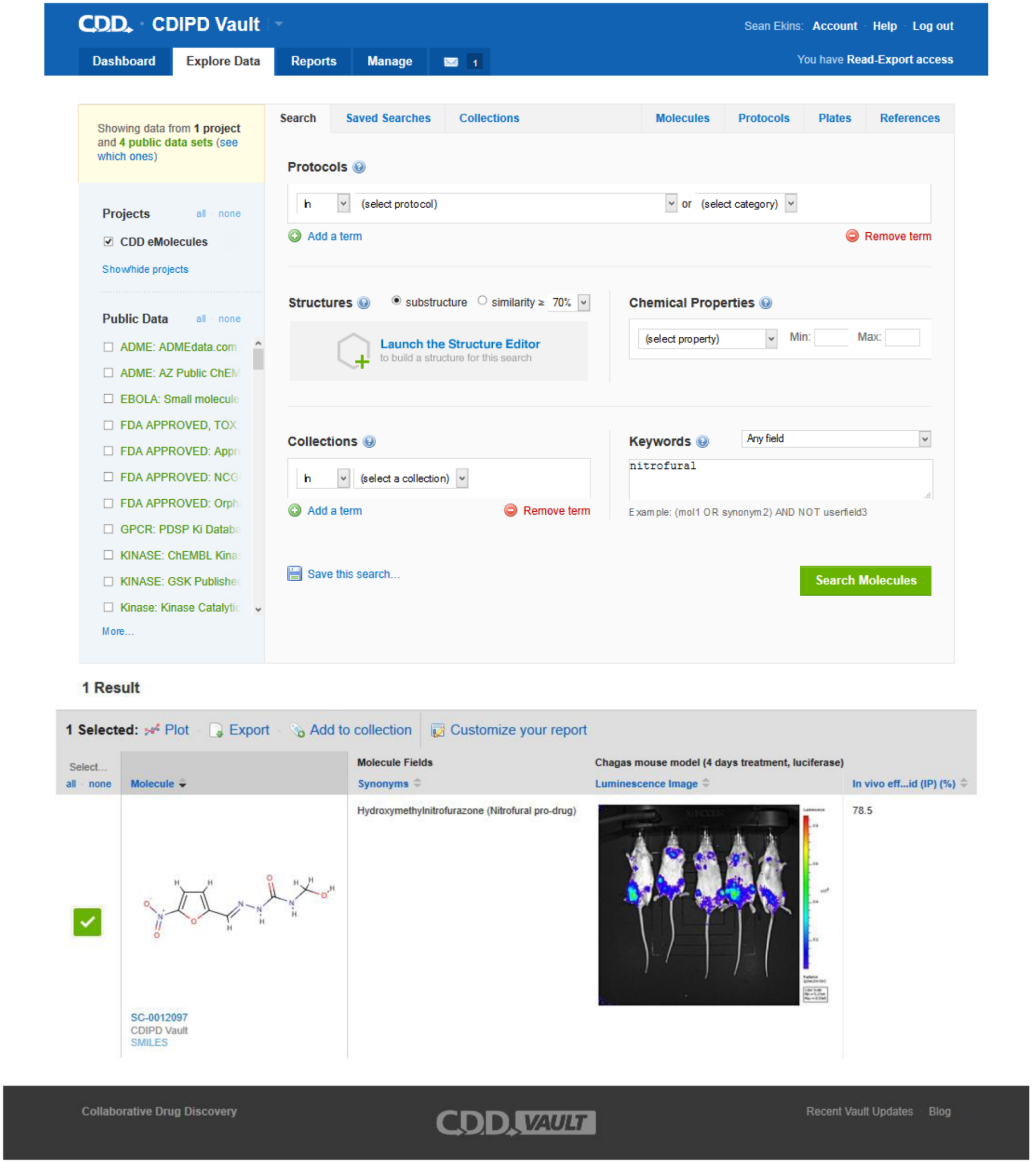
An example showing the CDD Vault for this collaboration, illustrating how the structures and biology data can be securely shared.

This study made wide use of public datasets in CDD as well as the collaborative sharing of data in the CDD Vault. We have also highlighted how the *in vivo* transgenic *T*.*cruzi* Brazil luc strain expressing firefly luciferase data can be stored in the software (Fig 3). These data will ultimately be made publically accessible in this format alongside the datasets we have already made public. In the process of this study we have curated *T*. *cruzi* data, constructed a Pathway Genome Data Base for *T*. *cruzi* (Fig 1), developed multiple Bayesian machine learning models, tested molecules *in vitro* and *in vivo* as well as proposed potential targets for one of the *in vivo* active compounds. In the process we have identified pyronaridine as having promising *in vivo* activity in the mouse model of Chagas disease. Future studies will evaluate efficacy in longer term models and identify the target or targets of this molecule. The approaches taken are broadly applicable to other NTDs and extend our prior work with *Mtb* [42,43,46,47,56–63]. Leveraging published data to create additional resources and models for either re-mining known or new datasets to suggest compounds that can be rapidly progressed all the way through to *in vivo* animal models, may lead to new clinical studies in a shorter time scale. There are many steps we could take to update our computational models such as incorporating the current data and using other machine learning algorithms. If we can in future narrow down the list of possible targets computationally as well and accelerate experimental target validation that will also be of importance. The combination of computational and experimental approaches represents a multistep workflow (S8 Fig) that was undertaken in this study that could be applicable in any NTD drug discovery project. Efforts to automate, streamline and learn from the resulting data would further increase the efficiency of the approach we have described.

## Supporting Information

S1 Table Leave-out 50% x 100 fold for Chagas dose response and cytotoxicity Bayesian model. (DOCX)

S2 Table Primary and dose response results. Infection Ratio: number of infected cells divided by the total number of cells. Primary screening was done in duplicate, thus the two values for infection ratio (at 10 **μ**M).
(DOCX)

S1 Fig Broad Chagas (T Cruzi) dose response: good features from FCFP_6. (DOCX)

S2 Fig Broad Chagas (T Cruzi) dose response: bad features from FCFP_6. (DOCX)

S3 Fig Broad Chagas (T Cruzi) dose response and cytotox: good features from FCFP_6. (DOCX)

S4 Fig Broad Chagas (T Cruzi) dose response and cytotox: bad features from FCFP_6. (DOCX)

S5 Fig *In vivo* efficacy of test compounds in a 4-day mouse model of infection by transgenic *T*.*cruzi* Brazil luc strain expressing firefly luciferase. (DOCX)

S6 Fig Similarity search with pyronaridine in literature dataset curated on Chagas Disease in CDD. (DOCX)

S7 Fig A similarity search on ChEMBL using the MMDS (Molecular Materials Informatics, Inc. Montreal Canada) app. One of the most similar compounds was quinacrine an antimalarial with the target trypanothione disulfide reductase.
(DOCX)

S8 Fig Workflow in this project with discrete steps which could be automated. (DOCX)

S1 Dataset Dose response Discovery Studio Bayesian model files include model XML, protocol XML and molecule SDF. (ZIP)

S2 Dataset Dose response and cytotoxicity Discovery Studio Bayesian model files include model XML, protocol XML and molecule SDF. (ZIP)
