# Supplementary material for: Machine Learning Models and Pathway Genome Data Base for Trypanosoma cruzi Drug Discovery
Source: PLoS Negl Trop Dis. 2015 Jun 26;9(6):e0003878. doi: 10.1371/journal.pntd.0003878 (PMC4482694; doi:10.1371/journal.pntd.0003878)
Supplement: S1 Table — (DOCX) [file pntd.0003878.s001.docx]

**S1 Table. Leave-out 50% x 100 fold for Chagas dose response and cytotoxicity Bayesian model.**

|  |  |  |  |  |
| --- | --- | --- | --- | --- |
| **External ROC** | **Internal ROC** | **Concordance (%)** | **Specificity**  **(%)** | **Sensitivity (%)** |
| 0.79 ± 0.01 | 0.80 ± 0.01 | 73.48 ± 1.05 | 79.08 ± 3.73 | 65.68 ± 3.89 |
