# Supplementary material for: Machine Learning Models and Pathway Genome Data Base for Trypanosoma cruzi Drug Discovery
Source: PLoS Negl Trop Dis. 2015 Jun 26;9(6):e0003878. doi: 10.1371/journal.pntd.0003878 (PMC4482694; doi:10.1371/journal.pntd.0003878)
Supplement: S2 Table — Infection Ratio: number of infected cells divided by the total number of cells. Primary screening was done in duplicate, thus the two values for infection ratio (at 10 μM). (DOCX) [file pntd.0003878.s002.docx]

**S2 Table. Primary and dose response results.** Infection Ratio: number of infected cells divided by the total number of cells. Primary screening was done in duplicate, thus the two values for infection ratio (at 10 **μ**M).

| **Molecule Name** | **Structure** | **Synonyms** | **Infection Ratio** | **EC50 (μM)** | **EC90 (μM)** | **Hill slope** |
| --- | --- | --- | --- | --- | --- | --- |
| SC-0005695 |    \|  \| \| --- \| | (±)-Verapamil hydrochloride, 715730, SC-0011762 | 0.019458858, 0.016758591 | 0.0383 | 0.143 | 1.67 |
| SC-0011799 |    \|  \| \| --- \| | 29781612, Pyronaridine | 0, 0 | 0.225 | 0.665 | 2.03 |
| SC-0011742 |    \|  \| \| --- \| | 511176, Furazolidone | 7.712766E-03, 8.769277E-03 | 0.257 | 0.563 | 2.81 |
| SC-0011496 | \|  \| \| --- \| | 501337, SC-0011777, Tetrandine | 0, 0 | 0.508 | 1.57 | 1.95 |
| SC-0011754 |    \|  \| \| --- \| | 511054  Nitrofural | 0.012269939, 0.010091743 | 0.775 | 6.98 | 1 |
| SC-0011833 |    \|  \| \| --- \| | 511499 | 4.020101E-03, 7.333683E-03 | 1.04 | 5.36 | 1.34 |
| SC-0011790 |    \|  \| \| --- \| | 739223 | 0.018524756, 0.017738359 | 1.68 | > 10.0 | 0.743 |
| SC-0011752 |    \|  \| \| --- \| | 2735992 | 0.028985507, 0.011235955 | 1.69 | 2.44 | 6.04 |
| SC-0011772 |    \|  \| \| --- \| | 539712 | 0.012753502, 6.218333E-03 | 2.44 | 5.14 | 2.95 |
| SC-0011803 |    \|  \| \| --- \| | 876406 | 0.027158313, 0.038105727 | 2.72 | 3.75 | 6.86 |
| SC-0011796 |    \|  \| \| --- \| | 515815 | 7.481297E-03, 8.14901E-03 | 4.94 | > 10.0 | 2.18 |
| SC-0011806 |    \|  \| \| --- \| | 6856294 | 0.266666667, 0.275438596 | > 10.0 | > 10.0 | -1.3 |
| SC-0011810 |    \|  \| \| --- \| | 1989320 | 0.038045505, 0.043723811 | > 10.0 | > 10.0 | 1.15 |
| SC-0011814 |    \|  \| \| --- \| | 532231 | 0.078934625, 0.094926611 | > 10.0 | > 10.0 | 0.69 |
| SC-0011466 |    \|  \| \| --- \| | 48203181, SC-0011821, cetylpyridinium chloride | 0.037414966, 0.03654485 | > 10.0 | > 10.0 | -1.22 |
| SC-0011829 |    \|  \| \| --- \| | 206844054 | 0.092783505, 0.123542274 | > 10.0 | > 10.0 | 0.539 |
| SC-0011739 |    \|  \| \| --- \| | 33575177 | 8.5531E-03, 0.021348514 | > 10.0 | > 10.0 | 16.6 |
| SC-0011801 |    \|  \| \| --- \| | 1936034 | 0.192489851, 0.172903673 | | |  |
| SC-0011817 |    \|  \| \| --- \| | 1985496 | 0.064082886, 0.05501562 | | |  |
| SC-0011798 |    \|  \| \| --- \| | 518121 | 0.073818375, 0.099157253 | | |  |
| SC-0011832 |    \|  \| \| --- \| | 481205 | 0.079290369, 0.068433916 | | |  |
| SC-0011757 |    \|  \| \| --- \| | 475504 | 0.10454893, 0.108330534 | | |  |
| SC-0011793 |    \|  \| \| --- \| | 975809 | 0.049488055, 0.065289913 | | |  |
| SC-0011783 |    \|  \| \| --- \| | 511411 | 0.062519249, 0.085890622 | | |  |
| SC-0011826 |    \|  \| \| --- \| | 529362 | 0.052091946, 0.072594356 | | |  |
| SC-0011820 |    \|  \| \| --- \| | 538640 | 0.087166602, 0.095264117 | | |  |
| SC-0011751 |    \|  \| \| --- \| | 729687 | 0.076673497, 0.117647059 | | |  |
| SC-0011768 |    \|  \| \| --- \| | 531385 | 0.105658709, 0.110689197 | | |  |
| SC-0011766 |    \|  \| \| --- \| | 538440 | 0.104049392, 0.095416093 | | |  |
| SC-0011773 |    \|  \| \| --- \| | 593947 | 0.092101004, 0.09862543 | | |  |
| SC-0011811 |    \|  \| \| --- \| | 741109 | 0.023306773, 0.026873857 | | |  |
| SC-0011825 |    \|  \| \| --- \| | 873594 | 0.075423105, 0.073272129 | | |  |
| SC-0011738 |    \|  \| \| --- \| | 509347 | 0.056658879, 0.057180851 | | |  |
| SC-0011760 |    \|  \| \| --- \| | 44537757 | 0.118173988, 0.09408826 | | |  |
| SC-0011787 |    \|  \| \| --- \| | 489450 | 0.077537233, 0.08559322 | | |  |
| SC-0011763 |    \|  \| \| --- \| | 489259 | 0.096113074, 0.110482588 | | |  |
| SC-0011750 |    \|  \| \| --- \| | 531050 | 0.047833515, 0.092283951 | | |  |
| SC-0011594 |    \|  \| \| --- \| | 513312, GNF-00-0910-3412-7, NVP-AKI399-NX-4, SC-0011755 | 0.07839051, 0.085458537 | | |  |
| SC-0011771 |    \|  \| \| --- \| | 626322 | 0.075085883, 0.095469799 | | |  |
| SC-0011487 |    \|  \| \| --- \| | 592188, Pargyline hydrochloride, SC-0011774 | 0.07607362, 0.08506474 | | |  |
| SC-0011795 |    \|  \| \| --- \| | 538596 | 0.086387, 0.102647868 | | |  |
| SC-0011831 |    \|  \| \| --- \| | 901874 | 0.043439873, 0.063611491 | | |  |
| SC-0011780 |    \|  \| \| --- \| | 594529 | 0.050304878, 0.08496306 | | |  |
| SC-0011784 |    \|  \| \| --- \| | 711729 | 0.078268711, 0.097461237 | | |  |
| SC-0011747 |    \|  \| \| --- \| | 529222 | 0.113586957, 0.134515321 | | |  |
| SC-0011578 |    \|  \| \| --- \| | 533320, GNF-00-0910-3411-6, PKF042-567-NX-6, SC-0011759 | 0.12035124, 0.071439811 | | |  |
| SC-0011786 |    \|  \| \| --- \| | 206844082 | 0.079000335, 0.094679038 | | |  |
| SC-0011794 |    \|  \| \| --- \| | 988007 | 0.077545383, 0.068086656 | | |  |
| SC-0011741 |    \|  \| \| --- \| | 886566 | 0.07013383, 0.04071397 | | |  |
| SC-0011765 |    \|  \| \| --- \| | 902391 | 0.082668086, 0.089789061 | | |  |
| SC-0011792 |    \|  \| \| --- \| | 29541115 | 0.051118211, 0.083416087 | | |  |
| SC-0011809 |    \|  \| \| --- \| | 535324 | 0.082362205, 0.086597938 | | |  |
| SC-0011761 |    \|  \| \| --- \| | 207290596 | 0.047838997, 0.039863096 | | |  |
| SC-0011812 |    \|  \| \| --- \| | 206903720 | 0.051139422, 0.049784982 | | |  |
| SC-0011823 |    \|  \| \| --- \| | 594920 | 0.026855572, 0.03051106 | | |  |
| SC-0011769 |    \|  \| \| --- \| | 497513 | 0.099270321, 0.136662286 | | |  |
| SC-0011748 |    \|  \| \| --- \| | 45576197 | 0.057455995, 0.071752793 | | |  |
| SC-0011745 |    \|  \| \| --- \| | 206842881 | 0.144903881, 0.102429297 | | |  |
| SC-0011822 |    \|  \| \| --- \| | 1096780 | 0.058916568, 0.05049239 | | |  |
| SC-0011807 |    \|  \| \| --- \| | 711328 | 0.08160075, 0.084018112 | | |  |
| SC-0011781 |    \|  \| \| --- \| | 594468 | 0.09339934, 0.093515358 | | |  |
| SC-0011791 |    \|  \| \| --- \| | 32952277 | 0.111828751, 0.092341633 | | |  |
| SC-0011749 |    \|  \| \| --- \| | 902145 | 0.101623887, 0.118987342 | | |  |
| SC-0011819 |    \|  \| \| --- \| | 1254510 | 0.061541027, 0.081500647 | | |  |
| SC-0011802 |    \|  \| \| --- \| | 874033 | 0.146865159, 0.178231772 | | |  |
| SC-0011805 |    \|  \| \| --- \| | 594160 | 0.079886686, 0.067870385 | | |  |
| SC-0011737 |    \|  \| \| --- \| | 476877 | 0.084826133, 0.051508203 | | |  |
| SC-0011746 |    \|  \| \| --- \| | 46510709 | 0.117904762, 0.124646959 | | |  |
| SC-0011828 |    \|  \| \| --- \| | 2736593 | 0.051312414, 0.069783313 | | |  |
| SC-0011797 |    \|  \| \| --- \| | 206904131 | 0.111758989, 0.118572928 | | |  |
| SC-0011758 |    \|  \| \| --- \| | 1935912 | 0.072871697, 0.153160622 | | |  |
| SC-0011788 |    \|  \| \| --- \| | 35779513 | 0.049784268, 0.063105998 | | |  |
| SC-0011753 |    \|  \| \| --- \| | 2253190 | 0.074377457, 0.123972603 | | |  |
| SC-0011764 |    \|  \| \| --- \| | 35775543 | 0.086123476, 0.103568658 | | |  |
| SC-0011756 |    \|  \| \| --- \| | 2736517 | 0.070663094, 0.1152222 | | |  |
| SC-0011789 |    \|  \| \| --- \| | 594064 | 0.064027939, 0.084274953 | | |  |
| SC-0011770 |    \|  \| \| --- \| | 902298 | 0.1117598, 0.151923077 | | |  |
| SC-0011785 |    \|  \| \| --- \| | 6884964 | 0.068929685, 0.116246001 | | |  |
| SC-0011782 |    \|  \| \| --- \| | 30513626 | 0.077220727, 0.113482941 | | |  |
| SC-0011740 |    \|  \| \| --- \| | 504178 | 0.076170376, 0.055576208 | | |  |
| SC-0011775 |    \|  \| \| --- \| | 29370212 | 0.10306354, 0.100158144 | | |  |
| SC-0011743 |    \|  \| \| --- \| | 6879996 | 0.060392018, 0.048125451 | | |  |
| SC-0011808 |    \|  \| \| --- \| | 31240769 | 0.114944356, 0.130626317 | | |  |
| SC-0011818 |    \|  \| \| --- \| | 498094 | 0.060895522, 0.086980712 | | |  |
| SC-0011744 |    \|  \| \| --- \| | 1935924 | 0.163581286, 0.179317203 | | |  |
| SC-0011816 |    \|  \| \| --- \| | 2736631 | 0.059011802, 0.058238636 | | |  |
| SC-0011804 |    \|  \| \| --- \| | 4826672 | 0.090265022, 0.086489253 | | |  |
| SC-0011824 |    \|  \| \| --- \| | 520704 | 0.048384424, 0.086551047 | | |  |
| SC-0011800 |    \|  \| \| --- \| | 595565 | 0.084427456, 0.1322794 | | |  |
| SC-0011815 |    \|  \| \| --- \| | 1934024 | 0.06360601, 0.129328487 | | |  |
| SC-0011767 |    \|  \| \| --- \| | 4704194 | 0.100881262, 0.110520095 | | |  |
| SC-0011776 |    \|  \| \| --- \| | 206845378 | 0.081705787, 0.089192591 | | |  |
| SC-0011827 |    \|  \| \| --- \| | 1406771 | 0.074934839, 0.068834459 | | |  |
| SC-0011830 |    \|  \| \| --- \| | 4775492 | 0.064033955, 0.054005722 | | |  |
| SC-0011779 |    \|  \| \| --- \| | 27249335 | 0.118130766, 0.076069213 | | |  |
| SC-0011778 |    \|  \| \| --- \| | 29547719 | 0.103939501, 0.102104443 | | |  |
| SC-0011813 |    \|  \| \| --- \| | 46012953 | 0.085594406, 0.072580645 | | |  |
