# Supplementary material for: Machine Learning Models and Pathway Genome Data Base for Trypanosoma cruzi Drug Discovery
Source: PLoS Negl Trop Dis. 2015 Jun 26;9(6):e0003878. doi: 10.1371/journal.pntd.0003878 (PMC4482694; doi:10.1371/journal.pntd.0003878)
Supplement: S1 Fig — (DOCX) [file pntd.0003878.s003.docx]

**S1 Fig. Broad Chagas (T Cruzi) dose response: good features from FCFP_6**

| \| 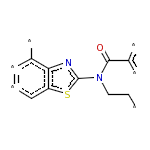 \| \| --- \| \| G1: 1151340232 56 out of 57 good Bayesian Score: 0.754 \| | \| 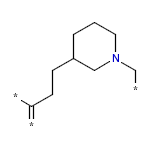 \| \| --- \| \| G2: -1031377555 27 out of 27 good Bayesian Score: 0.750 \| | \| 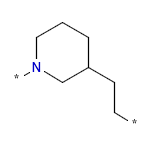 \| \| --- \| \| G3: -553669137 27 out of 27 good Bayesian Score: 0.750 \| | \| 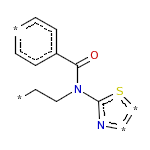 \| \| --- \| \| G4: 284237667 48 out of 49 good Bayesian Score: 0.748 \| | \| 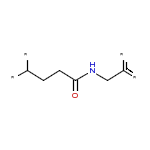 \| \| --- \| \| G5: 193414534 25 out of 25 good Bayesian Score: 0.747 \| |
| --- | --- | --- | --- | --- | --- | --- | --- | --- | --- | --- | --- | --- | --- | --- |
| \| 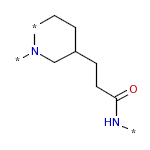 \| \| --- \| \| G6: -551668701 25 out of 25 good Bayesian Score: 0.747 \| | \| 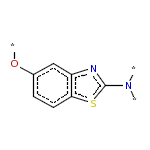 \| \| --- \| \| G7: -1214801979 25 out of 25 good Bayesian Score: 0.747 \| | \| 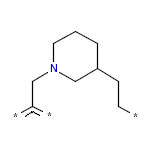 \| \| --- \| \| G8: 84111596 23 out of 23 good Bayesian Score: 0.743 \| | \| 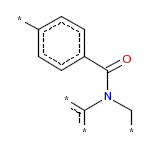 \| \| --- \| \| G9: -1799269324 38 out of 39 good Bayesian Score: 0.737 \| | \| 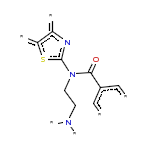 \| \| --- \| \| G10: 776700060 19 out of 19 good Bayesian Score: 0.734 \| |
| \| 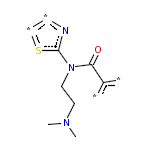 \| \| --- \| \| G11: 1566208234 19 out of 19 good Bayesian Score: 0.734 \| | \| 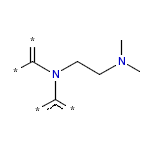 \| \| --- \| \| G12: -356513920 19 out of 19 good Bayesian Score: 0.734 \| | \| 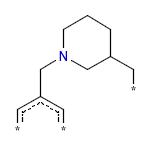 \| \| --- \| \| G13: -1541471493 73 out of 77 good Bayesian Score: 0.724 \| | \| 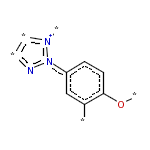 \| \| --- \| \| G14: -301872638 16 out of 16 good Bayesian Score: 0.724 \| | \| 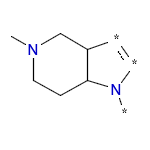 \| \| --- \| \| G15: 675763288 16 out of 16 good Bayesian Score: 0.724 \| |
| \| 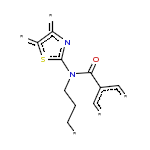 \| \| --- \| \| G16: 774560441 30 out of 31 good Bayesian Score: 0.724 \| | \| 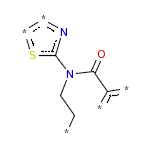 \| \| --- \| \| G17: -609539162 58 out of 61 good Bayesian Score: 0.724 \| | \| 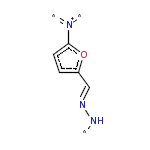 \| \| --- \| \| G18: -1808404930 56 out of 59 good Bayesian Score: 0.721 \| | \| 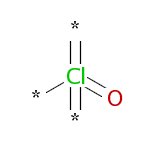 \| \| --- \| \| G19: 1873107836 15 out of 15 good Bayesian Score: 0.720 \| | \| 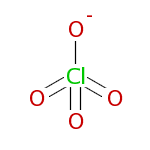 \| \| --- \| \| G20: -6220344 15 out of 15 good Bayesian Score: 0.720 \| |
