# Supplementary material for: Machine Learning Models and Pathway Genome Data Base for Trypanosoma cruzi Drug Discovery
Source: PLoS Negl Trop Dis. 2015 Jun 26;9(6):e0003878. doi: 10.1371/journal.pntd.0003878 (PMC4482694; doi:10.1371/journal.pntd.0003878)
Supplement: S2 Fig — (DOCX) [file pntd.0003878.s004.docx]

**S2 Fig. Broad Chagas (T Cruzi) dose response: bad features from FCFP_6**

| \| 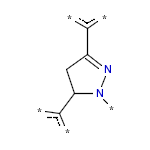 \| \| --- \| \| B1: 453075031 0 out of 23 good Bayesian Score: -2.435 \| | \| 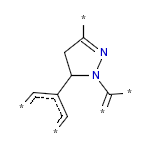 \| \| --- \| \| B2: -975093520 0 out of 19 good Bayesian Score: -2.262 \| | \| 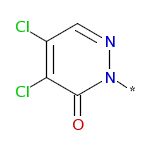 \| \| --- \| \| B3: -1782262991 0 out of 18 good Bayesian Score: -2.214 \| | \| 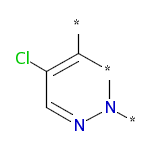 \| \| --- \| \| B4: 1485918631 0 out of 18 good Bayesian Score: -2.214 \| | \| 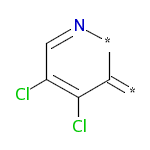 \| \| --- \| \| B5: -1110304493 0 out of 18 good Bayesian Score: -2.214 \| |
| --- | --- | --- | --- | --- | --- | --- | --- | --- | --- | --- | --- | --- | --- | --- |
| \| 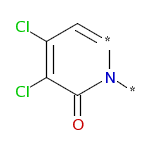 \| \| --- \| \| B6: -1116653357 0 out of 18 good Bayesian Score: -2.214 \| | \| 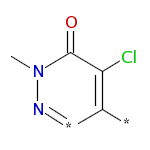 \| \| --- \| \| B7: 357154323 0 out of 18 good Bayesian Score: -2.214 \| | \| 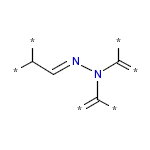 \| \| --- \| \| B8: 1585170049 0 out of 18 good Bayesian Score: -2.214 \| | \| 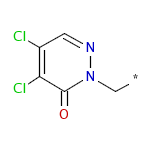 \| \| --- \| \| B9: 1249923161 0 out of 16 good Bayesian Score: -2.109 \| | \| 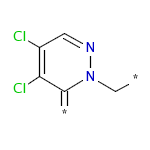 \| \| --- \| \| B10: 1108944866 0 out of 16 good Bayesian Score: -2.109 \| |
| \| 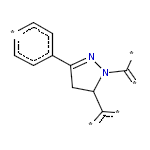 \| \| --- \| \| B11: -36630029 0 out of 14 good Bayesian Score: -1.993 \| | \| 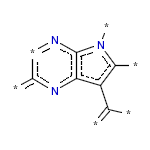 \| \| --- \| \| B12: 1796123205 0 out of 13 good Bayesian Score: -1.929 \| | \| 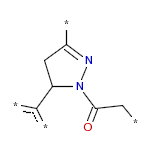 \| \| --- \| \| B13: 433286102 0 out of 13 good Bayesian Score: -1.929 \| | \| 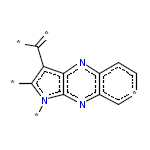 \| \| --- \| \| B14: 1559056674 0 out of 13 good Bayesian Score: -1.929 \| | \| 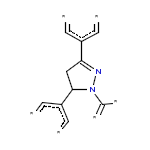 \| \| --- \| \| B15: 676207562 0 out of 13 good Bayesian Score: -1.929 \| |
| \| 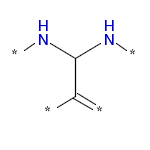 \| \| --- \| \| B16: -109786778 0 out of 13 good Bayesian Score: -1.929 \| | \| 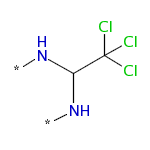 \| \| --- \| \| B17: 32983469 0 out of 12 good Bayesian Score: -1.861 \| | \| 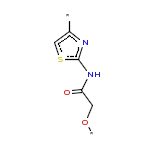 \| \| --- \| \| B18: -144598603 0 out of 12 good Bayesian Score: -1.861 \| | \| 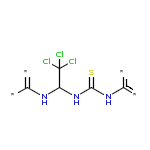 \| \| --- \| \| B19: 632156824 0 out of 12 good Bayesian Score: -1.861 \| | \| 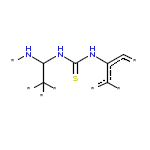 \| \| --- \| \| B20: -2082572339 0 out of 12 good Bayesian Score: -1.861 \| |
