# Supplementary material for: Machine Learning Models and Pathway Genome Data Base for Trypanosoma cruzi Drug Discovery
Source: PLoS Negl Trop Dis. 2015 Jun 26;9(6):e0003878. doi: 10.1371/journal.pntd.0003878 (PMC4482694; doi:10.1371/journal.pntd.0003878)
Supplement: S3 Fig — (DOCX) [file pntd.0003878.s005.docx]

**S3 Fig. Broad Chagas (T Cruzi) dose response and cytotox: good features from FCFP_6**

| \| 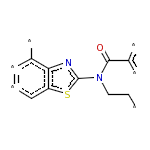 \| \| --- \| \| G1: 1151340232 56 out of 57 good Bayesian Score: 0.836 \| | \| 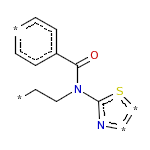 \| \| --- \| \| G2: 284237667 48 out of 49 good Bayesian Score: 0.830 \| | \| 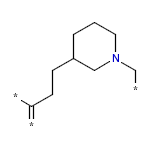 \| \| --- \| \| G3: -1031377555 27 out of 27 good Bayesian Score: 0.829 \| | \| 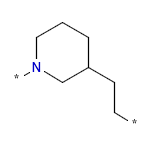 \| \| --- \| \| G4: -553669137 27 out of 27 good Bayesian Score: 0.829 \| | \| 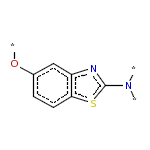 \| \| --- \| \| G5: -1214801979 25 out of 25 good Bayesian Score: 0.825 \| |
| --- | --- | --- | --- | --- | --- | --- | --- | --- | --- | --- | --- | --- | --- | --- |
| \| 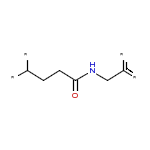 \| \| --- \| \| G6: 193414534 25 out of 25 good Bayesian Score: 0.825 \| | \| 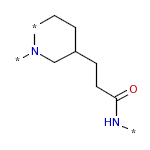 \| \| --- \| \| G7: -551668701 25 out of 25 good Bayesian Score: 0.825 \| | \| 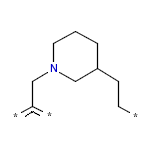 \| \| --- \| \| G8: 84111596 23 out of 23 good Bayesian Score: 0.821 \| | \| 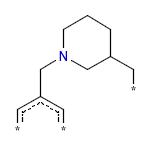 \| \| --- \| \| G9: -1541471493 74 out of 77 good Bayesian Score: 0.821 \| | \| 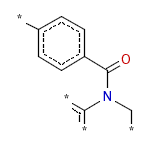 \| \| --- \| \| G10: -1799269324 38 out of 39 good Bayesian Score: 0.818 \| |
|  |  |  |  |  |
| \| 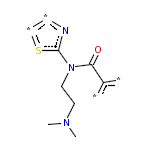 \| \| --- \| \| G11: 1566208234 19 out of 19 good Bayesian Score: 0.810 \| | \| 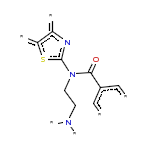 \| \| --- \| \| G12: 776700060 19 out of 19 good Bayesian Score: 0.810 \| | \| 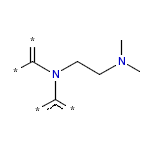 \| \| --- \| \| G13: -356513920 19 out of 19 good Bayesian Score: 0.810 \| | \| 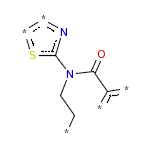 \| \| --- \| \| G14: -609539162 58 out of 61 good Bayesian Score: 0.806 \| | \| 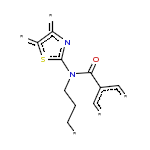 \| \| --- \| \| G15: 774560441 30 out of 31 good Bayesian Score: 0.803 \| |
| \| 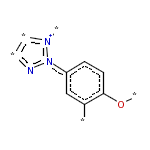 \| \| --- \| \| G16: -301872638 16 out of 16 good Bayesian Score: 0.798 \| | \| 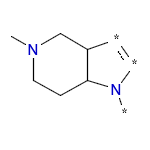 \| \| --- \| \| G17: 675763288 16 out of 16 good Bayesian Score: 0.798 \| | \| 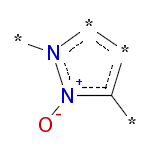 \| \| --- \| \| G18: -1715686892 28 out of 29 good Bayesian Score: 0.798 \| | \| 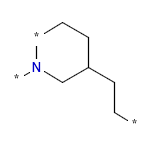 \| \| --- \| \| G19: 1422550102 27 out of 28 good Bayesian Score: 0.795 \| | \| 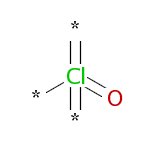 \| \| --- \| \| G20: 1873107836 15 out of 15 good Bayesian Score: 0.794 \| |
