# Supplementary material for: Machine Learning Models and Pathway Genome Data Base for Trypanosoma cruzi Drug Discovery
Source: PLoS Negl Trop Dis. 2015 Jun 26;9(6):e0003878. doi: 10.1371/journal.pntd.0003878 (PMC4482694; doi:10.1371/journal.pntd.0003878)
Supplement: S4 Fig — (DOCX) [file pntd.0003878.s006.docx]

**S4 Fig. Broad Chagas (T Cruzi) dose response and cytotox: bad features from FCFP_6**

| \| 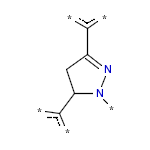 \| \| --- \| \| B1: 453075031 0 out of 23 good Bayesian Score: -2.357 \| | \| 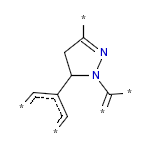 \| \| --- \| \| B2: -975093520 0 out of 19 good Bayesian Score: -2.186 \| | \| 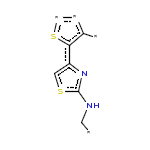 \| \| --- \| \| B3: 1474313868 0 out of 19 good Bayesian Score: -2.186 \| | \| 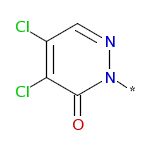 \| \| --- \| \| B4: -1782262991 0 out of 18 good Bayesian Score: -2.138 \| | \| 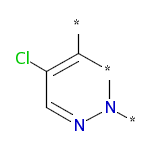 \| \| --- \| \| B5: 1485918631 0 out of 18 good Bayesian Score: -2.138 \| |
| --- | --- | --- | --- | --- | --- | --- | --- | --- | --- | --- | --- | --- | --- | --- |
| \| 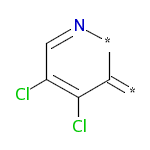 \| \| --- \| \| B6: -1110304493 0 out of 18 good Bayesian Score: -2.138 \| | \| 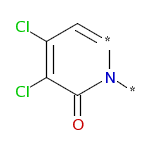 \| \| --- \| \| B7: -1116653357 0 out of 18 good Bayesian Score: -2.138 \| | \| 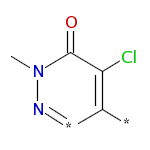 \| \| --- \| \| B8: 357154323 0 out of 18 good Bayesian Score: -2.138 \| | \| 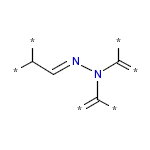 \| \| --- \| \| B9: 1585170049 0 out of 18 good Bayesian Score: -2.138 \| | \| 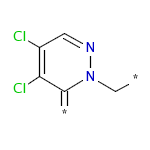 \| \| --- \| \| B10: 1108944866 0 out of 16 good Bayesian Score: -2.035 \| |
| \| 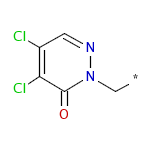 \| \| --- \| \| B11: 1249923161 0 out of 16 good Bayesian Score: -2.035 \| | \| 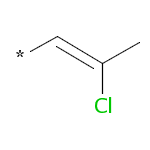 \| \| --- \| \| B12: 501530171 1 out of 34 good Bayesian Score: -2.024 \| | \| 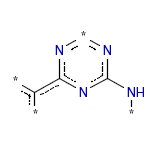 \| \| --- \| \| B13: -1775135141 0 out of 15 good Bayesian Score: -1.979 \| | \| 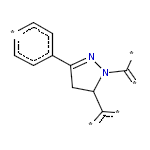 \| \| --- \| \| B14: -36630029 0 out of 14 good Bayesian Score: -1.920 \| | \| 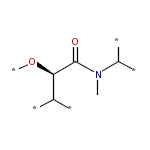 \| \| --- \| \| B15: -950109695 0 out of 14 good Bayesian Score: -1.920 \| |
| \| 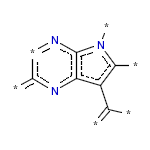 \| \| --- \| \| B16: 1796123205 0 out of 13 good Bayesian Score: -1.857 \| | \| 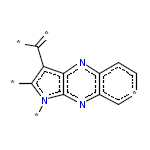 \| \| --- \| \| B17: 1559056674 0 out of 13 good Bayesian Score: -1.857 \| | \| 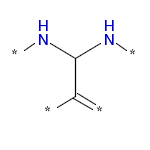 \| \| --- \| \| B18: -109786778 0 out of 13 good Bayesian Score: -1.857 \| | \| 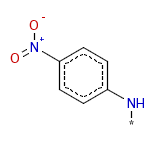 \| \| --- \| \| B19: 76171628 0 out of 13 good Bayesian Score: -1.857 \| | \| 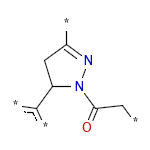 \| \| --- \| \| B20: 433286102 0 out of 13 good Bayesian Score: -1.857 \| |
