# Supplementary material for: Machine Learning Models and Pathway Genome Data Base for Trypanosoma cruzi Drug Discovery
Source: PLoS Negl Trop Dis. 2015 Jun 26;9(6):e0003878. doi: 10.1371/journal.pntd.0003878 (PMC4482694; doi:10.1371/journal.pntd.0003878)

**S5 Fig. *In vivo* efficacy of test compounds in a 4-day mouse model of infection by transgenic *T.cruzi* Brazil luc strain expressing firefly luciferase**


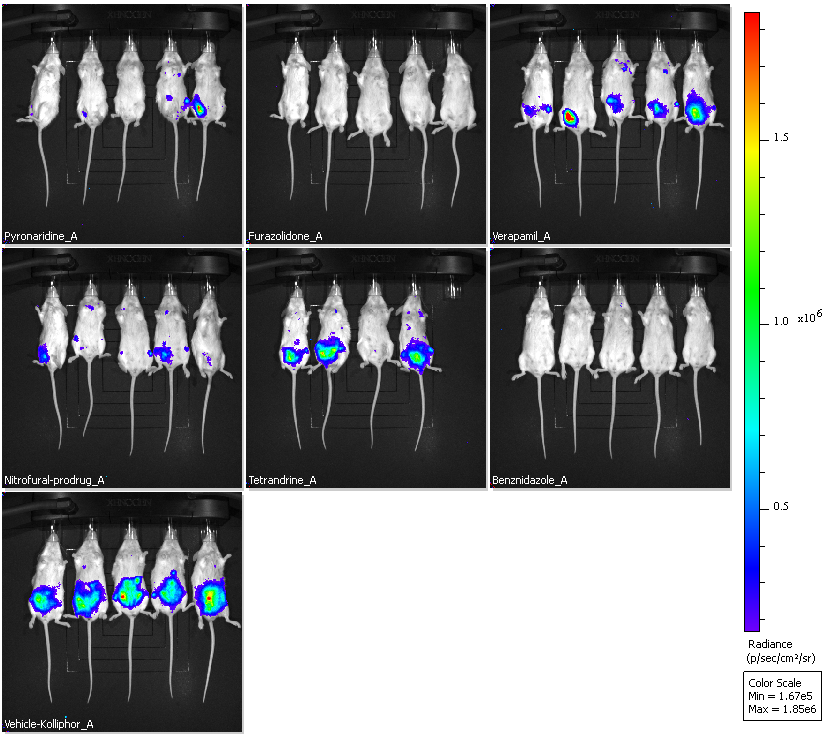

Supplement: S5 Fig — (DOCX) [file pntd.0003878.s007.docx]
