# Supplementary material for: Machine Learning Models and Pathway Genome Data Base for Trypanosoma cruzi Drug Discovery
Source: PLoS Negl Trop Dis. 2015 Jun 26;9(6):e0003878. doi: 10.1371/journal.pntd.0003878 (PMC4482694; doi:10.1371/journal.pntd.0003878)

**S6 Fig. Similarity search with pyronaridine in literature dataset curated on Chagas Disease in CDD**


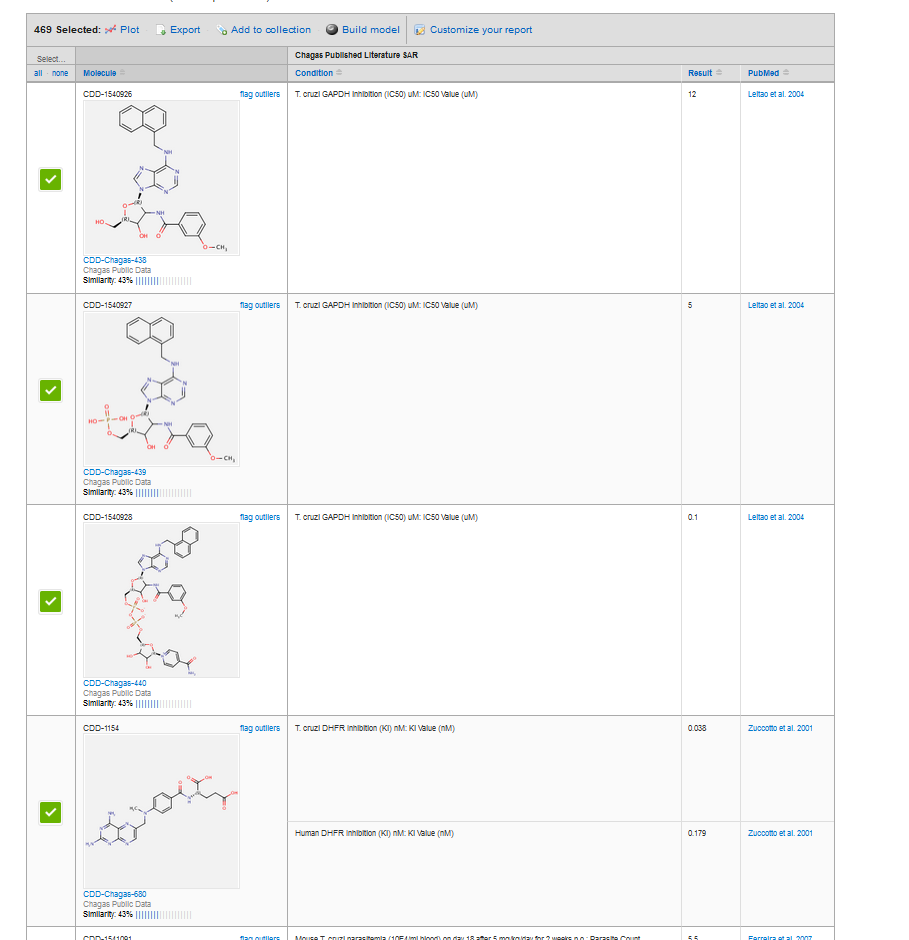

Supplement: S6 Fig — (DOCX) [file pntd.0003878.s008.docx]
