# Supplementary material for: Machine Learning Models and Pathway Genome Data Base for Trypanosoma cruzi Drug Discovery
Source: PLoS Negl Trop Dis. 2015 Jun 26;9(6):e0003878. doi: 10.1371/journal.pntd.0003878 (PMC4482694; doi:10.1371/journal.pntd.0003878)
Supplement: S7 Fig — One of the most similar compounds was quinacrine an antimalarial with the target trypanothione disulfide reductase. (DOCX) [file pntd.0003878.s009.docx]

**S7 Fig. A similarity search on ChEMBL using the MMDS (Molecular Materials Informatics, Inc. Montreal Canada) app.** One of the most similar compounds was quinacrine an antimalarial with the target trypanothione disulfide reductase.

**
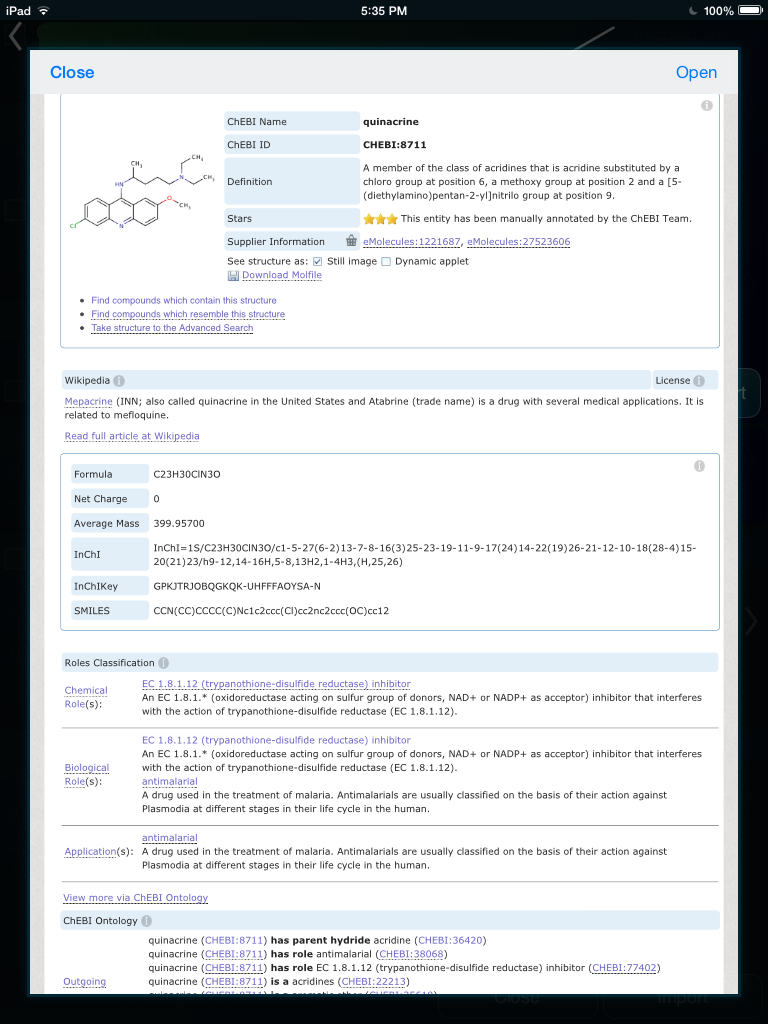
**
