# Supplementary figures and images for: Machine Learning Models and Pathway Genome Data Base for Trypanosoma cruzi Drug Discovery
Source: PLoS Negl Trop Dis. 2015 Jun 26;9(6):e0003878. doi: 10.1371/journal.pntd.0003878 (PMC4482694; doi:10.1371/journal.pntd.0003878)

**S8 Fig. Workflow in this project with discrete steps which could be automated.**


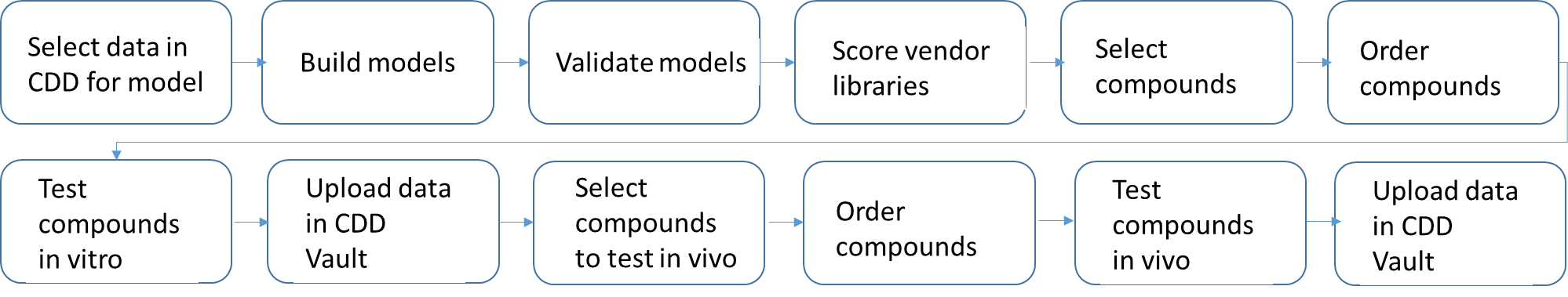

Supplement: S8 Fig — (DOCX) [file pntd.0003878.s010.docx]
